# The use of cystatin C to inhibit epithelial–mesenchymal transition and morphological transformation stimulated by transforming growth factor-β

**DOI:** 10.1186/bcr1312

**Published:** 2005-08-23

**Authors:** Jonathan P Sokol, Jason R Neil, Barbara J Schiemann, William P Schiemann

**Affiliations:** 1 Program in Cell Biology, Department of Pediatrics, National Jewish Medical and Research Center, Denver, CO, USA

## Abstract

**Introduction:**

Transforming growth factor-β (TGF-β) is a potent suppressor of mammary epithelial cell (MEC) proliferation and is thus an inhibitor of mammary tumor formation. Malignant MECs typically evolve resistance to TGF-β-mediated growth arrest, enhancing their proliferation, invasion, and metastasis when stimulated by TGF-β. Recent findings suggest that therapeutics designed to antagonize TGF-β signaling may alleviate breast cancer progression, thereby improving the prognosis and treatment of breast cancer patients. We identified the cysteine protease inhibitor cystatin C (CystC) as a novel TGF-β type II receptor antagonist that inhibits TGF-β binding and signaling in normal and cancer cells. We hypothesized that the oncogenic activities of TGF-β, particularly its stimulation of mammary epithelial–mesenchymal transition (EMT), can be prevented by CystC.

**Method:**

Retroviral infection was used to constitutively express CystC or a CystC mutant impaired in its ability to inhibit cathepsin protease activity (namely Δ14CystC) in murine NMuMG MECs and in normal rat kidney (NRK) fibroblasts. The effect of recombinant CystC administration or CystC expression on TGF-β stimulation of NMuMG cell EMT *in vitro *was determined with immunofluorescence to monitor rearrangements of actin cytoskeletal architecture and E-cadherin expression. Soft-agar growth assays were performed to determine the effectiveness of CystC in preventing TGF-β stimulation of morphological transformation and anchorage-independent growth in NRK fibroblasts. Matrigel invasion assays were performed to determine the ability of CystC to inhibit NMuMG and NRK motility stimulated by TGF-β.

**Results:**

CystC and Δ14CystC both inhibited NMuMG cell EMT and invasion stimulated by TGF-β by preventing actin cytoskeletal rearrangements and E-cadherin downregulation. Moreover, both CystC molecules completely antagonized TGF-β-mediated morphological transformation and anchorage-independent growth of NRK cells, and inhibited their invasion through synthetic basement membranes. Both CystC and Δ14CystC also inhibited TGF-β signaling in two tumorigenic human breast cancer cell lines.

**Conclusion:**

Our findings show that TGF-β stimulation of initiating metastatic events, including decreased cell polarization, reduced cell–cell contact, and elevated cell invasion and migration, are prevented by CystC treatment. Our findings also suggest that the future development of CystC or its peptide mimetics hold the potential to improve the therapeutic response of human breast cancers regulated by TGF-β.

## Introduction

Oncogenic epithelial–mesenchymal transitions (EMTs) comprise a complex array of gene expression and repression that elicits tumor metastasis in localized carcinomas [1,2]. The acquisition of metastatic phenotypes by dedifferentiated tumors is the most lethal facet of cancer and is the leading cause of cancer-related death [3,4]. Transforming growth factor-β (TGF-β) is a powerful tumor suppressor that normally represses these processes by prohibiting epithelial cell proliferation, and by creating a cell microenvironment that inhibits epithelial cell motility, invasion, and metastasis [5,6]. Carcinogenesis often subverts the tumor-suppressing function of TGF-β, thereby endowing TGF-β with oncogenic activities that promote the growth and spread of developing tumors, including the initiation and stabilization of tumor EMT [1,2,5-7]. The duality of TGF-β in both suppressing and promoting cancer development was observed originally with transgenic TGF-β1 expression in mouse keratinocytes, which initially suppressed benign skin tumor formation before promoting malignant conversion and spindle cell carcinoma generation [8]. More recently, TGF-β signaling was shown to inhibit the tumorigenicity of normal, premalignant, and malignant breast epithelial cells, while stimulating that of highly invasive and metastatic breast cancer cells [9]. Fundamental gaps exist in our knowledge of how malignant cells overcome the cytostatic actions of TGF-β and of how TGF-β stimulates the progression of developing tumors. Indeed, these knowledge gaps have prevented science and medicine from developing treatments effective in antagonizing TGF-β oncogenicity in progressing cancers, particularly those of the breast.

The ability of TGF-β to induce cancer growth and metastasis suggests that developing therapeutics to antagonize and/or circumvent TGF-β signaling may prove effective in treating metastatic malignancies, perhaps by preventing the stimulation of EMT by TGF-β. Cystatin C (CystC) is a small, ubiquitously expressed cysteine protease inhibitor found in nearly all bodily fluids [10,11]. By inactivating cathepsin protease activities, CystC reduces bone resorption, neutrophil chemotaxis, and tissue inflammation, and also inhibits cancer cell invasion [10]. We showed recently that CystC not only inhibits cathepsin-mediated invasion but also antagonizes TGF-β signaling in normal and cancer cells by interacting physically with the TGF-β type II receptor (TβR-II), thereby preventing TGF-β binding [12]. Thus, CystC is a novel TβR-II antagonist that may prove useful in blocking the oncogenic activities of TGF-β, particularly its ability to stimulate EMT. In the present study we tested this hypothesis by measuring the ability of CystC to antagonize the oncogenic activities of TGF-β in two established *in vitro *models of cancer progression: first, EMT of normal murine NMuMG mammary epithelial cells (MECs), and second, morphological transformation and anchorage-independent growth of normal rat kidney (NRK) fibroblasts. We show that CystC significantly decreased TGF-β stimulation of EMT and morphological transformation in mammary epithelial cells and kidney fibroblasts, respectively. We show further that CystC significantly antagonized TGF-β signaling in two tumorigenic human breast cancer cell lines. Thus, by antagonizing TGF-β signaling and preventing its stimulation of EMT, CystC may represent a novel TGF-β chemopreventive agent effective in neutralizing the tumor-promoting activities of TGF-β and its stimulation of tumor metastasis.

## Materials and methods

### Recombinant CystC expression and purification

The synthesis of bacterial expression vectors encoding human CystC or Δ14CystC fused to the carboxy terminus of glutathione S-transferase (GST), and their purification from transformed *Escherichia coli*, were described previously [12].

### Retroviral CystC expression

The creation of bicistronic retroviral vectors (namely pMSCV-IRES–GFP, where GFP stands for green fluorescent protein) encoding human CystC or Δ14CystC was described previously [12]. Mouse NMuMG, rat NRK kidney fibroblasts, and human MDA-MB-231 and MCF10A-CA1a breast cancer cells were infected overnight with control (pMSCV-IRES–GFP), CystC, or Δ14CystC retroviral supernatants produced by EcoPac2 retroviral packaging cells (Clontech) as described [12]. Cells expressing GFP were isolated and collected 48 hours later on a MoFlo cell sorter (Cytomation), and were subsequently expanded to yield stable polyclonal populations of control, CystC-expressing, or Δ14CystC-expressing cells. The expression and secretion of recombinant CystC proteins by individual infected cell lines were monitored by immunoblotting conditioned medium with anti-CystC antibodies as described [12].

### Immunofluorescence studies

The ability of TGF-β to alter actin cytoskeletal architecture and E-cadherin expression was monitored essentially as described [13]. In brief, control, CystC-expressing, or Δ14CystC-expressing NMuMG cells were allowed to adhere overnight to glass coverslips in 24-well plates (50,000 cells per well). The cells were stimulated the following day with TGF-β1 (5 ng/ml) for 0 to 36 hours at 37°C. In some experiments, control NMuMG cells were stimulated with TGF-β1 in the absence or presence of 10 μg/ml recombinant GST, GST–CystC, or GST–Δ14CystC. On completion of agonist stimulation, the cells were washed in ice-cold PBS and immediately fixed in 3.7% formaldehyde. After extensive washing in PBS, the cells were blocked in PBS supplemented with 1.5% FBS, followed by incubation with rhodamine–phalloidin (0.25 μM). Alternatively, the cells were blocked in goat γ-globulin (200 μg/ml; Jackson Immunoresearch) before the detection of E-cadherin by sequential incubations with monoclonal anti-E-cadherin (1:50 dilution; BD Bioscience), followed by biotinylated goat anti-mouse antibody (5 μg/ml; Jackson Immunoresearch), and finally by Alexa-streptavidin (1.2 μg/ml; Molecular Probes). Images were captured on a Nikon Diaphot microscope.

The ability of TGF-β to alter E-cadherin expression was also monitored by immunoprecipitating E-cadherin from buffer H/Triton (1.5 mM EGTA, 50 mM β-glycerophosphate, 1 mM DTT, 0.2 mM Na_3_VO_4_, 1 mM benzamidine, 10 μg/ml Leupeptin, 10 μg/ml aprotinin, and 1% Triton X-100; pH 7.3) [14]. NMuMG whole cell extracts (250 μg per tube), followed by immunoblotting with anti-E-cadherin antibodies (1:1000 dilution). In other experiments, an aliquot of NMuMG whole cell extract was removed before immunoprecipitation of E-cadherin and used to control for differences in protein loading by immunoblotting for extracellular signal-related kinase 1/2. Both assay protocols yielded similar results.

### Cell biological assays

The effect CystC or Δ14CystC on various MECs (such as NMuMG, MCF10A-CA1a, or MDA-MB-231) or NRK cell activities was determined as follows: first, cell proliferation with a [^3^H]thymidine incorporation assay as described [14,15]; second, cell invasion with a modified Boyden-chamber assay and Matrigel matrices as described [12,15]; and third, gene expression with pSBE-luciferase and p3TP-luciferase reporter gene assays as described [12,14].

In addition, the effect of recombinant GST, CystC, or Δ14CystC on Smad2 phosphorylation stimulated by TGF-β was determined by allowing MDA-MB-231 cells (100,000 cells per well) to adhere overnight to 24-well plates. The following morning, the cells were washed twice in ice-cold PBS and incubated in serum-free medium supplemented with 25 μg/ml recombinant GST, CystC, or Δ14CystC for 2 hours at 37°C. Afterward, the cells were stimulated with TGF-β1 (1 ng/ml) for 30 min at 37°C, and subsequently were subjected to phospho-Smad2 immunoblot analyses as described [14].

### Soft agar assay

The growth of NRK cells in soft agar was performed as described [16]. In brief, duplicate cultures of control, CystC-expressing, or Δ14CystC-expressing NRK cells (10,000 cells per plate) were grown in 0.3% agar on a cushion of 0.6% agar in 35 mm plates. NRK cell growth in the absence or presence of 5 ng/ml TGF-β1 was allowed to proceed for 7 days, whereupon the number of colonies formed was quantified under a light microscope.

## Results

### CystC prevents TGF-β stimulation of EMT in NMuMG cells

The importance of EMT in promoting cancer progression and tumor metastasis is becoming increasingly apparent [1,2]. Although the formation and growth of early-stage tumors is normally suppressed by TGF-β, cancer progression typically enables TGF-β to stimulate the growth and metastasis of late-stage tumors, in part through its induction of EMT [2]. We recently identified the cysteine protease inhibitor CystC as a novel TβR-II antagonist [12]. Indeed, the physical association of CystC with TβR-II not only prevented TGF-β binding and, consequently, TGF-β signaling in normal and cancer cells, but it also inhibited their invasion through synthetic basement membranes [12]. Collectively, these findings led us to propose CystC as a novel chemopreventive agent capable of antagonizing the tumor-promoting activities of TGF-β in late-stage tumors, particularly the ability of TGF-β to induce EMT.

**Figure 1:**
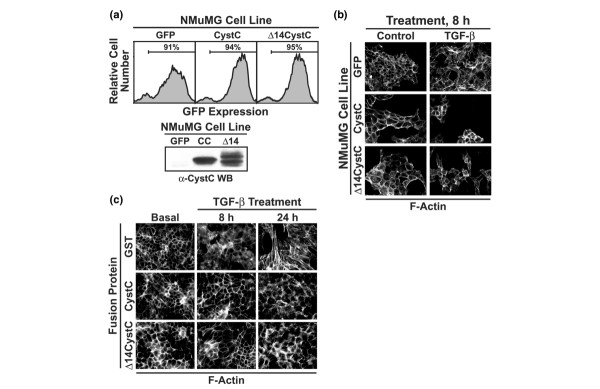
CystC and Δ14CystC inhibit actin cytoskeletal rearrangements stimulated by TGF-β in NMuMG cells. **(a) **NMuMG cells were infected with ecotropic retrovirus encoding either green fluorescent protein (GFP; control), CystC, or Δ14CystC, and subsequently were isolated by fluorescence-activated cell sorting for GFP expression to yield stable polyclonal populations of control, CystC-expressing, and Δ14CystC-expressing cells as indicated (upper panel). The expression and secretion of recombinant CystC proteins by infected NMuMG cells were monitored by immunoblotting conditioned medium with anti-CystC antibodies (lower panel). **(b) **Control, CystC-expressing, and Δ14CystC-expressing NMuMG cells were incubated in the absence or presence of transforming growth factor-β1 (TGF-β1; 5 ng/ml) for 8 hours, whereupon alterations in actin cytoskeletal architecture were revealed by direct rhodamine–phalloidin immunofluorescence. Shown are representative images from a single experiment that was repeated twice with identical results. **(c) **NMuMG cells were stimulated with TGF-β1 (5 ng/ml) for 0 to 24 hours in the presence of either glutathione S-transferase (GST), GST–CystC, or GST–Δ14CystC (each at 10 μg/ml) as indicated. Afterward, altered actin cytoskeletal architecture was revealed by direct rhodamine–phalloidin immunofluorescence. Shown are representative images from a single experiment that was repeated once with identical results.

To test this hypothesis, we first overexpressed in NMuMG cells (Fig. 1a) either CystC or a CystC mutant impaired in its ability to inhibit cathepsin protease activity (namely Δ14CystC [12]) to determine whether they could antagonize TGF-β stimulation of EMT. As described previously [12], Δ14CystC protein migrates more slowly in SDS-PAGE than its wild-type counterpart, because of additional amino-terminal amino acids appended to Δ14CystC during the shuttling of its cDNA through the pSecTag vector. Unlike HT1080 and 3T3-L1 cells, the expression of Δ14CystC in NMuMG cells results in the production of two distinct Δ14CystC molecules (Fig. 1a). Although they are currently unknown, we suspect that the two recombinant Δ14CystC species observed in transduced NMuMG cells most probably result from differences in glycosylation [17,18]. Nonetheless, these stable populations of NMuMG cells were used to examine the effects of CystC or Δ14CystC on NMuMG cell EMT.

In contrast to the situation in developing tissues, inappropriate induction of EMT by TGF-β in adult tissues enhances tumorigenesis [2,19]. TGF-β stimulation of EMT is studied routinely in murine NMuMG MECs, which readily undergo EMT when treated with TGF-β [13,20,21]. Unstimulated NMuMG cells exhibited typical epithelial cuboidal morphology characterized by strong cortical and diffuse cytoplasmic actin staining (Fig. 1b). In response to TGF-β, NMuMG cells undergo transition into fibroblasts and exhibit distinct actin stress fibers emanating from focal adhesions (Fig. 1b). This NMuMG cell response to TGF-β was prevented by the overexpression of either CystC or Δ14CystC (Fig. 1b). Similarly, treatment of NMuMG cells with recombinant CystC or Δ14CystC prevented TGF-β stimulation of actin cytoskeletal reorganization at 8 hours, and significantly reduced this response at 24 hours (Fig. 1c).

**Figure 2:**
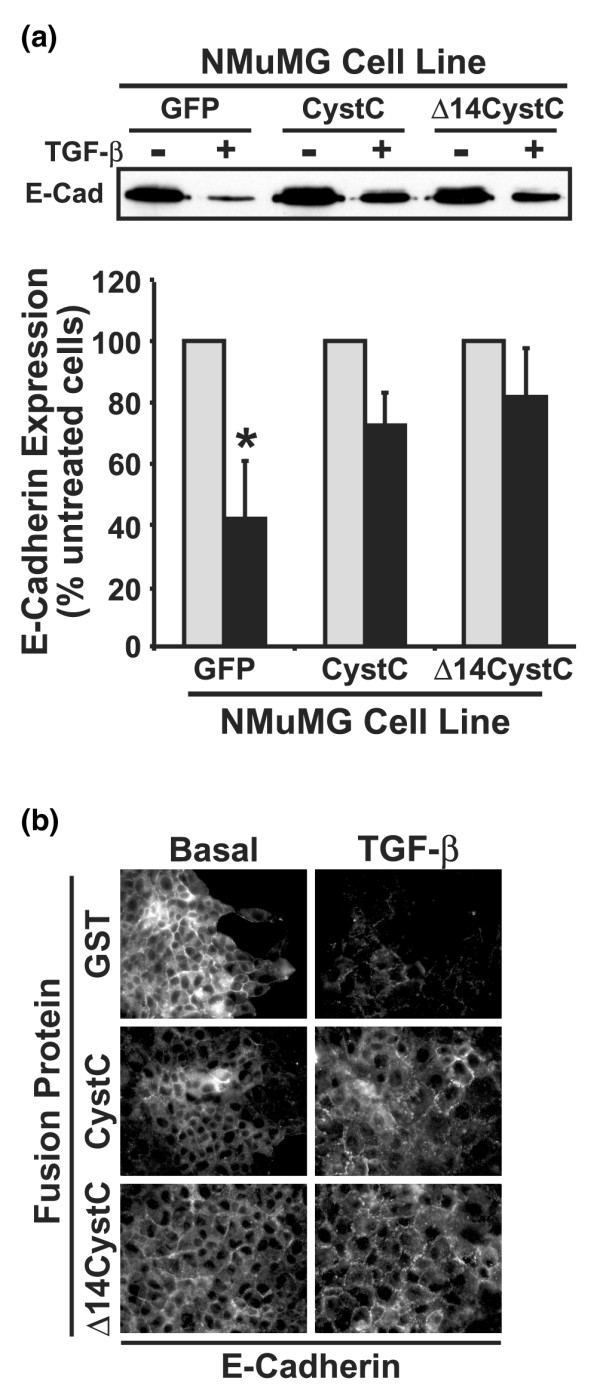
CystC and Δ14CystC antagonize E-cadherin downregulation stimulated by TGF-β in NMuMG cells. **(a) **Control, CystC-expressing, and Δ14CystC-expressing NMuMG cells were incubated in the absence or presence of transforming growth factor-β1 (TGF-β1; 5 ng/ml) for 36 hours, whereupon alterations in E-cadherin expression was monitored by immunoprecipitation and subsequent immunoblotting with anti-E-cadherin antibodies. The representative immunoblot depicts the downregulation of E-cadherin expression induced by TGF-β in NMuMG cells. The lower panel shows the alterations in E-cadherin expression (means ± SEM) induced by TGF-β relative to their untreated counterparts observed in three independent experiments. TGF-β significantly downregulated E-cadherin expression in NMuMG cells (*P < 0.05; Student's *t*-test). **(b) **NMuMG cells were stimulated with TGF-β1 (5 ng/ml) for 36 hours in the presence of glutathione S-transferase (GST) fusion proteins (10 μg/ml) as indicated. E-cadherin expression was monitored by indirect immunofluorescence with anti-E-cadherin antibodies. Shown are representative images from a single experiment that was repeated once with identical results.

**Figure 3:**
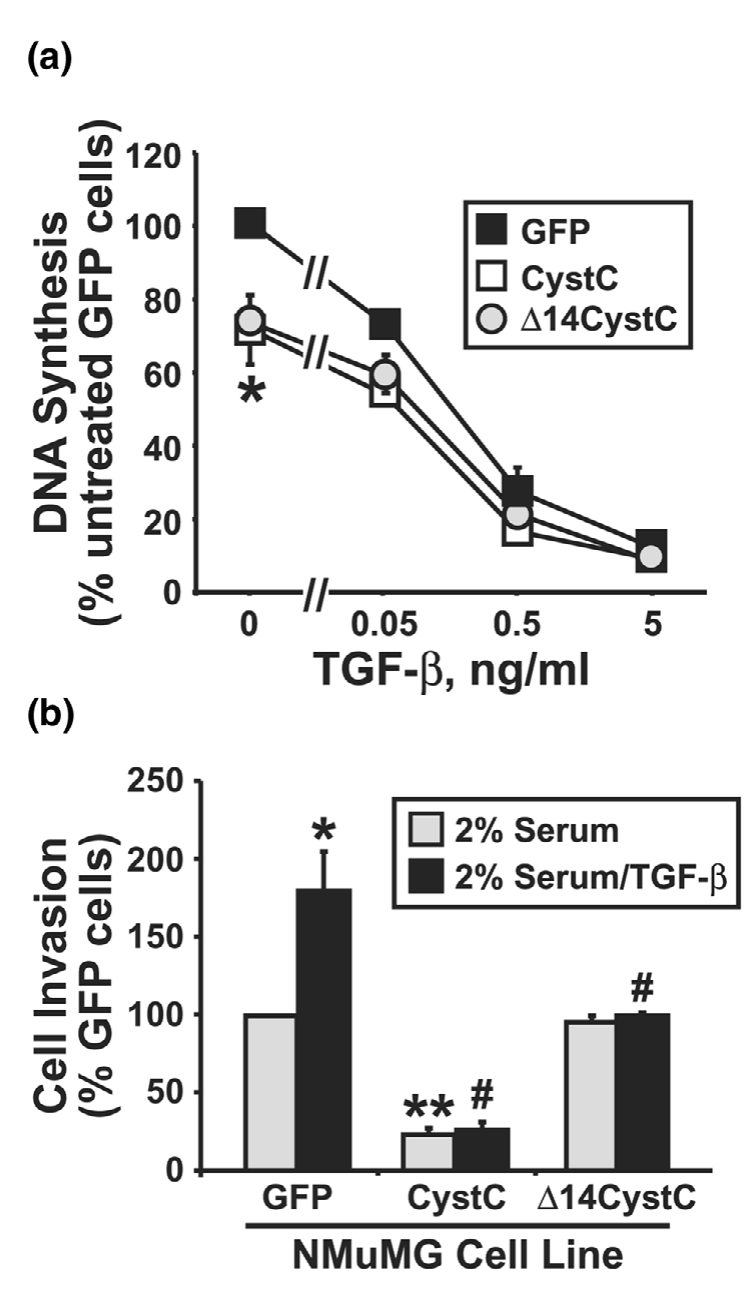
CystC and Δ14CystC inhibit NMuMG cell proliferation and invasion. **(a) **Control, CystC-expressing, or Δ14CystC-expressing NMuMG cells were incubated with increasing concentrations of transforming growth factor-β1 (TGF-β1; from 0 to 5 ng/ml) for 48 hours. Cellular DNA was radiolabeled with [^3^H]thymidine and quantified by scintillation counting. Data are means ± SEM for two independent experiments presented as the percentage [^3^H]thymidine incorporation normalized to untreated control cells. CystC and Δ14CystC both significantly decreased DNA synthesis in resting NMuMG cells (*P < 0.05; Student's *t*-test). **(b) **Control, CystC-expressing, and Δ14CystC-expressing NMuMG cells were allowed to invade through Matrigel matrices in the absence or presence of TGF-β1 (5 ng/ml) for 48 hours. Values are means ± SEM for three independent experiments presented as the percentage invasion relative to GFP-expressing NMuMG cells. TGF-β1 significantly enhanced NMuMG cell invasion (*P < 0.05; Student's *t*-test), a response that was inhibited significantly by CystC and Δ14CystC expression (^#^*P *< 0.05; Student's *t*-test). CystC expression also significantly inhibited tonic NMuMG cell invasion (*P < 0.05; Student's *t*-test).

TGF-β stimulation downregulates E-cadherin expression in MECs undergoing EMT [13]. We, too, find that TGF-β stimulation reduced NMuMG cell expression of E-cadherin and, more importantly, that CystC or Δ14CystC overexpression in or recombinant CystC or Δ14CystC treatment of NMuMG cells significantly attenuated the ability of TGF-β to downregulate E-cadherin expression (Fig. 2a, b). Figure 3a shows that TGF-β potently inhibited DNA synthesis in NMuMG cells. Quite surprisingly, CystC or Δ14CystC expression had little effect on the sensitivity of NMuMG cells to TGF-β-mediated growth arrest, despite the fact that both CystC molecules significantly inhibited tonic NMuMG cell proliferation (Fig. 3a). In addition, NMuMG cells undergoing EMT exhibit elevated invasion through synthetic basement membranes (Fig. 3b). We showed previously that, first, the invasion of 3T3-L1 cells through Matrigel matrices proceeds through pathways dependent on cathepsin and on TGF-β, and second, CystC abrogates both pathways, whereas Δ14CystC selectively blocks the TGF-β-dependent pathway [12]. Similarly to their actions on 3T3-L1 cells, the expression of CystC and Δ14CystC also inhibited and delineated cathepsin-dependent and TGF-β-dependent invasion in NMuMG cells (Fig. 3b). Collectively, these findings show that CystC and Δ14CystC do indeed effectively inhibit mammary cell EMT and invasion stimulated by TGF-β. Moreover, these findings suggest that when interacting with TβR-II, CystC alters the TGF-β receptor signaling complex in a manner that selectively inhibits some TGF-β functions (such as EMT) while leaving others (such as growth arrest) intact.

### CystC inhibits TGF-β signaling in human breast cancer cells

**Figure 4:**
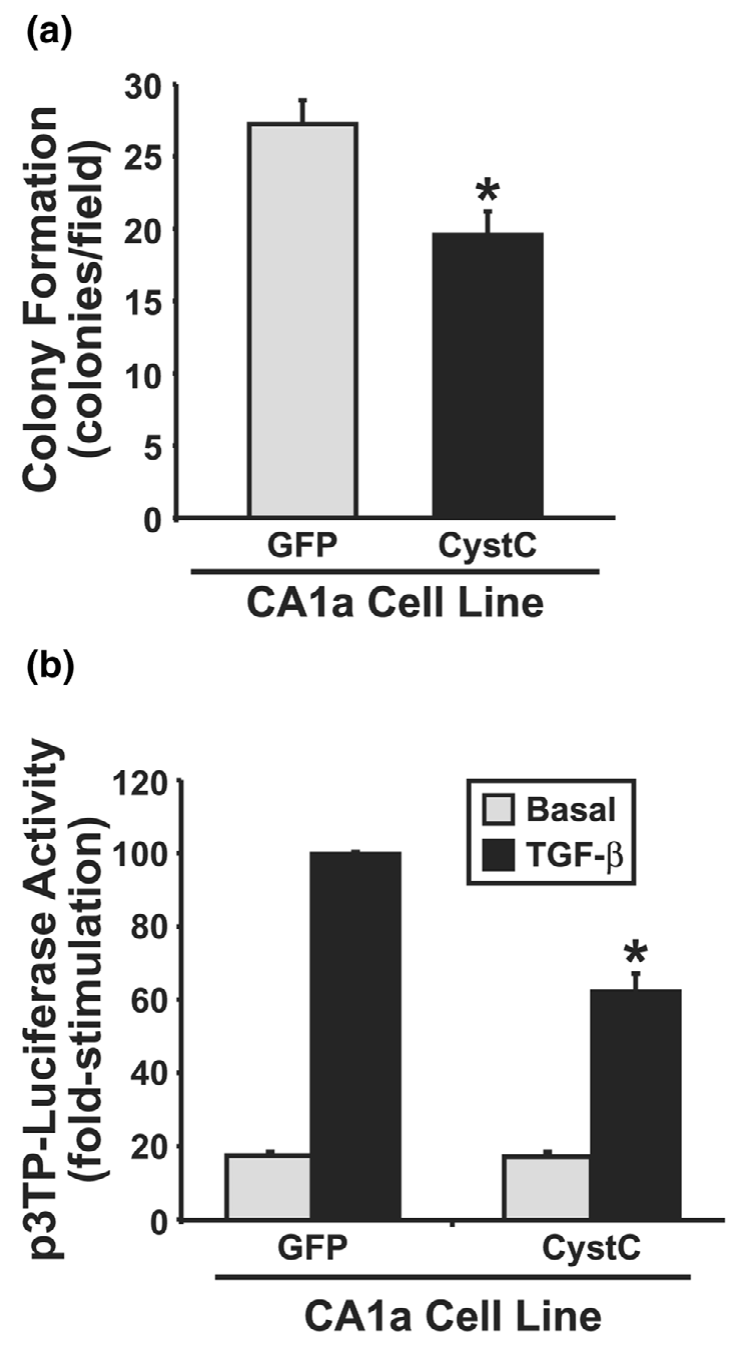
CystC antagonizes TGF-β signaling in human MCF10A-CA1a breast cancer cells. **(a) **Control and CystC-expressing MCF10A-CA1a cells were cultured in soft agar for 14 days, whereupon MCF10A-CA1a colony formation was quantified by light microscopy. Values are colony formation per microscope field (means ± SEM) observed in two independent experiments. CystC expression significantly reduced anchorage-independent growth of MCF10A-CA1a cells (*P < 0.05; Student's *t*-test). **(b) **Control and CystC-expressing MCF10A-CA1a cells were transiently transfected with p3TP-luciferase and pCMV-β-Gal cDNAs, and were subsequently stimulated with transforming growth factor-β1 (TGF-β1; 0.5 ng/ml) for 24 hours. Afterward, luciferase and β-Gal activities contained in detergent-solubilized cell extracts were measured. Values are luciferase activities (means ± SEM) observed in two independent experiments normalized to the maximal reporter gene expression stimulated by TGF-β in cells expressing green fluorescent protein. CystC expression significantly inhibited luciferase expression induced by TGF-β (*P < 0.05; Student's *t*-test).

Because NMuMG cells are non-tumorigenic and an incomplete model of breast cancer progression, we also determined whether CystC antagonizes TGF-β signaling in human breast cancer cells. To do so, we first stably expressed CystC in human MCF10A-CA1a breast cancer cells, whose metastatic activity is enhanced by TGF-β [9]. Given this fact, we hypothesized that TGF-β would stimulate the growth of MCF10A-CA1a cells in soft agar. In stark contrast, we found TGF-β stimulation to suppress the growth of MCF10A-CA1a cells in soft agar, a response that was unaffected by CystC expression (data not shown). However, CystC expression did significantly inhibit the anchorage-independent growth of MCF10A-CA1a cells (Fig. 4a), as well as significantly antagonized reporter gene expression stimulated by TGF-β (Fig. 4b).

**Figure 5:**
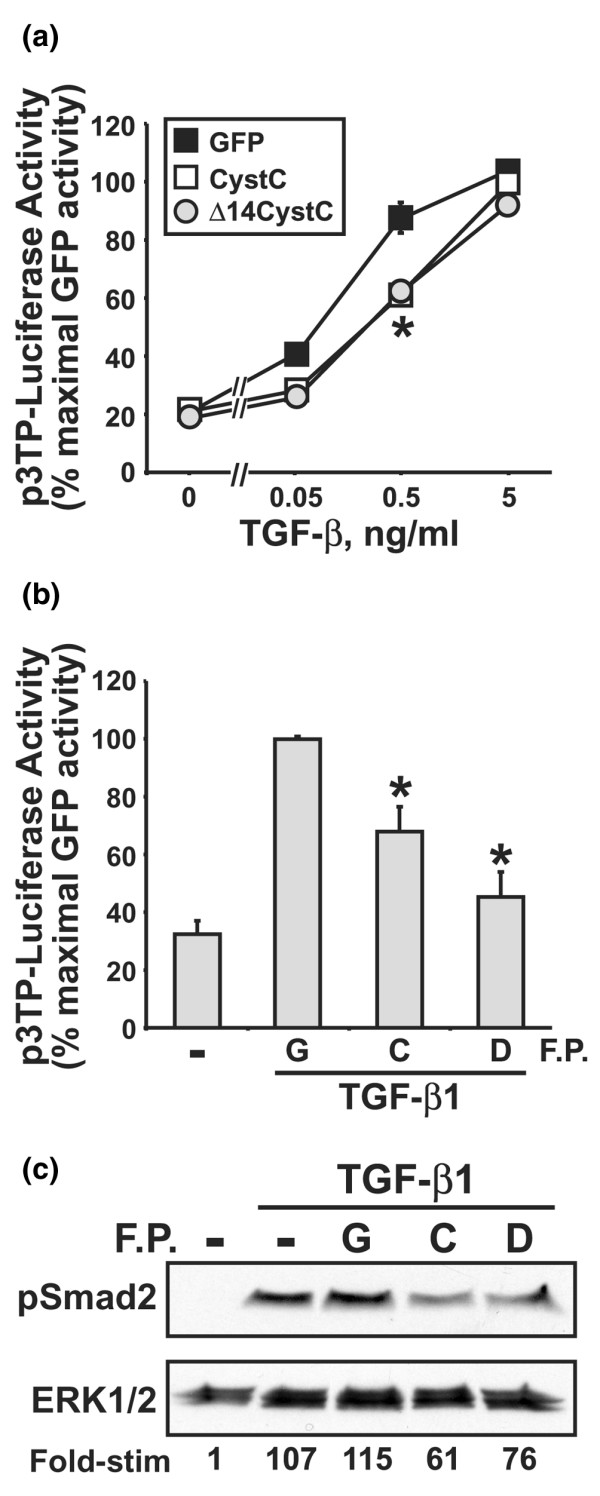
CystC and Δ14CystC inhibit TGF-β signaling in human MDA-MB-231 breast cancer cells. **(a) **Control, CystC-expressing, or Δ14CystC-expressing MDA-MB-231 cells were transiently transfected with p3TP-luciferase and pCMV-β-Gal cDNAs, and were subsequently stimulated with increasing concentrations of transforming growth factor-β1 (TGF-β1; from 0 to 5 ng/ml) for 24 hours. Afterward, luciferase and β-Gal activities contained in detergent-solubilized cell extracts were measured. Values are luciferase activities (means ± SEM) observed in two independent experiments normalized to maximal reporter gene expression in TGF-β-stimulated cells expressing green fluorescent protein. CystC and Δ14CystC both significantly inhibit reporter gene expression stimulated by TGF-β (*P < 0.05; Student's *t*-test). **(b) **MDA-MB-231 cells were transiently transfected with p3TP-luciferase and pCMV-β-Gal cDNAs. Afterward, the transfectants were treated with 25 μg/ml recombinant glutathione S-transferase (GST; G), CystC (C), or Δ14CystC (D) and immediately stimulated with TGF-β1 (1 ng/ml) for 24 hours before determination of the luciferase and β-Gal activities contained in detergent-solubilized cell extracts. Values are luciferase activities (means ± SEM) observed in four independent experiments normalized to maximal reporter gene expression stimulated by TGF-β in GST-treated cells. **(c) **MDA-MB-231 cells were treated with 25 μg/ml recombinant GST (G), CystC (C), or Δ14CystC (D) for 2 hours before their stimulation with TGF-β (1 ng/ml) for 30 min. Afterward, the activation status of Smad2 was determined by immunoblot analysis with phospho-specific Smad2 antibodies. Differences in protein loading were monitored by reprobing stripped membranes with antibodies against extracellular signal-related kinase 1. Data are from a representative experiment that was repeated once with similar results.

We also investigated whether the inhibitory effects of CystC on TGF-β signaling were unique to MCF10A-CA1a cells or were instead a more generalized inhibitory mechanism of TGF-β signaling in human breast cancers. As shown in Fig. 5a, overexpression of CystC or Δ14CystC in human MDA-MB-231 breast cancer cells significantly reduced reporter gene expression stimulated by moderate TGF-β concentrations (such as 0.5 ng/ml), an effect that was overcome by stimulation with supraphysiological TGF-β concentrations (such as 5 ng/ml). Accordingly, treatment of MDA-MB-231 cells with recombinant CystC or Δ14CystC significantly antagonized reporter gene expression induced by TGF-β (Fig. 5b) and inhibited its ability to stimulate Smad2 phosphorylation (Fig. 5c). Taken together, these findings establish CystC and its derivative Δ14CystC as novel TGF-β antagonists in human breast cancer cells.

### CystC prevents stimulation of morphological transformation in NRK cells by TGF-β

**Figure 6:**
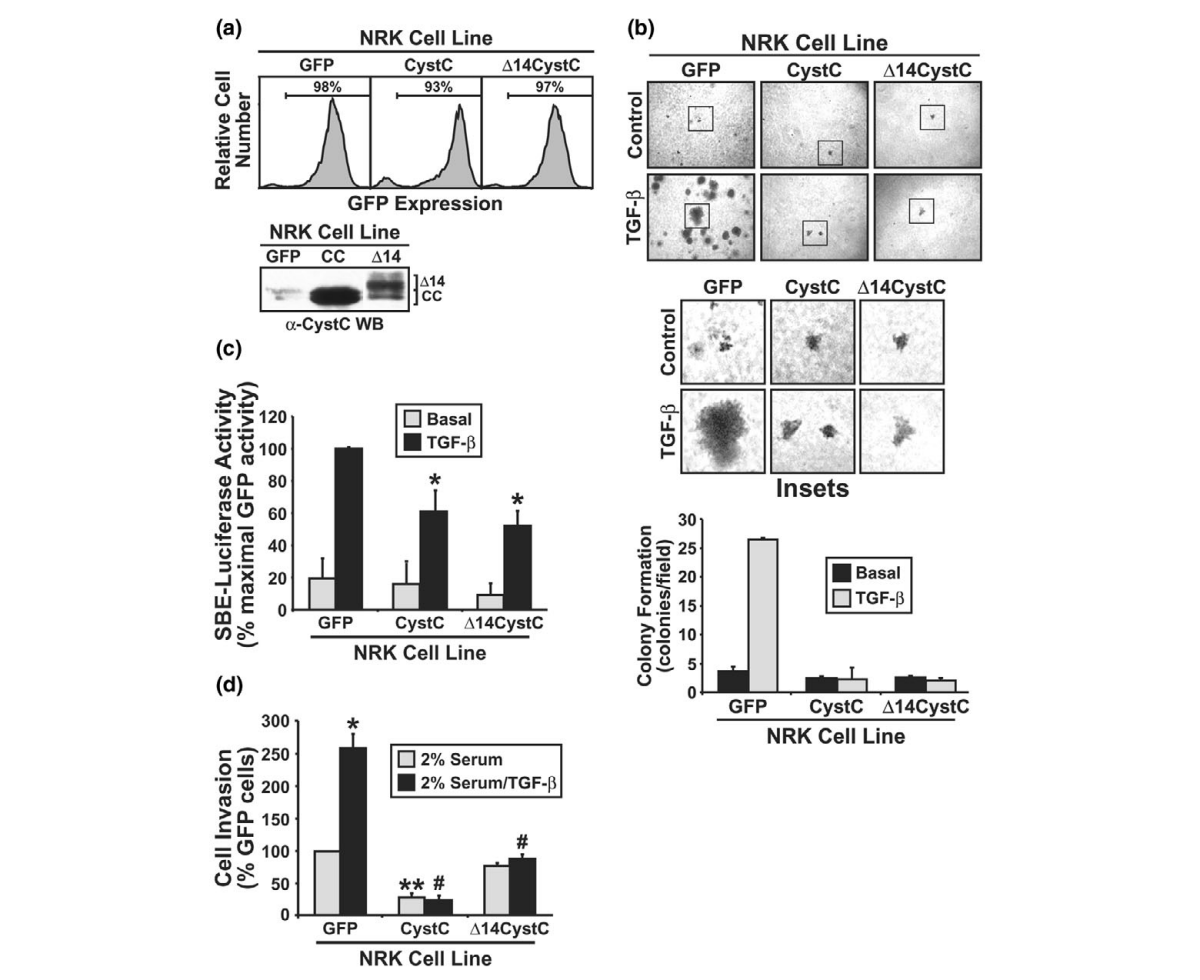
CystC and Δ14CystC inhibit NRK cell morphological transformation and anchorage-independent growth stimulated by TGF-β. **(a) **Normal rat kidney (NRK) fibroblasts were infected with control (namely pMSCV-IRES–GFP, where GFP stands for green fluorescent protein), CystC, or Δ14CystC retroviral supernatants as in Fig. 1a, and the resulting infected cells were isolated by GFP fluorescence on a MoFlo cell sorter 48 hours later. Shown in the upper panel are the GFP expression profiles of the resulting polyclonal populations. Also shown, in the lower panel, are recombinant CystC (CC) and Δ14CystC (Δ14) proteins present in conditioned medium of individual NRK populations and revealed by anti-CystC immunoblotting. **(b) **Control, CystC-expressing, and Δ14CystC-expressing NRK cells were cultured in soft agar in the absence or presence of transforming growth factor-β1 (TGF-β1; 5 ng/ml) for 7 days, whereupon NRK colony formation was quantified by light microscopy (upper panel). Middle panel, magnification of boxed regions. Lower panel, colony formation per microscope field (means ± SEM) observed in five independent experiments. **(c) **Control, CystC-expressing, or Δ14CystC-expressing NRK cells were transiently transfected with pSBE-luciferase and pCMV-β-Gal cDNAs, and were subsequently stimulated with TGF-β1 (5 ng/ml) for 24 hours. Afterward, luciferase and β-Gal activities contained in detergent-solubilized cell extracts were measured. Values are luciferase activities (means ± SEM) observed in three independent experiments normalized to maximal reporter gene expression induced by TGF-β in GFP-expressing cells. CystC expression significantly inhibited luciferase expression induced by TGF-β (*P < 0.05; Student's *t*-test). **(d) **Control, CystC-expressing, and Δ14CystC-expressing NRK cells were allowed to invade through Matrigel matrices in the absence or presence of TGF-β1 (5 ng/ml) for 48 hours. Values are means ± SEM for three independent experiments presented as the percentage invasion relative to GFP-expressing NRK cells. TGF-β1 significantly enhanced NRK cell invasion (*P < 0.05; Student's *t*-test). This TGF-β response was inhibited significantly by CystC and Δ14CystC expression (^#^*P *< 0.05; Student's *t*-test), whereas tonic NRK cell invasion was significantly inhibited only by CystC expression (*P < 0.05; Student's *t*-test). TGF-β1 significantly enhanced NMuMG cell invasion (*P < 0.05; Student's *t*-test), a response that was inhibited significantly by CystC and Δ14CystC expression (^#^*P *< 0.05; Student's *t*-test).

The loss of cell polarity and the ability of cancer cells to grow autonomously in an anchorage-independent manner are a hallmark of cancer [1,2]. TGF-β was originally described as a secreted factor that stimulates morphological transformation in rat NRK-49 kidney cells, leading to their acquisition of anchorage-independent growth in soft agar [22,23]. Thus, in addition to promoting EMT, TGF-β also enhances cancer progression by stimulating morphological transformation and anchorage-independent cell growth. Because CystC and Δ14CystC both eliminated EMT stimulated by TGF-β, we hypothesized that these TβR-II antagonists [12] would similarly inhibit NRK cell morphological transformation stimulated by TGF-β. We tested this hypothesis by infecting NRK cells with bicistronic retrovirus encoding either CystC or Δ14CystC (Fig. 6a) to determine their effects on NRK anchorage-independent growth stimulated by TGF-β. As expected, treatment with TGF-β enabled NRK cells to grow in an anchorage-independent manner when cultured in soft agar (Fig. 6b). Similar to their inhibitory activities in NMuMG and breast cancer cells, retroviral-mediated expression of CystC or Δ14CystC in NRK cells prevented their morphological transformation stimulated by TGF-β (Fig. 6b), as well as inhibiting Smad2/3-mediated reporter gene expression (Fig. 6c) and cell invasion induced by TGF-β (Fig. 6d). Thus, in addition to preventing TGF-β stimulation of EMT and inhibiting TGF-β signaling in breast cancer cells, CystC and Δ14CystC also antagonize morphological transformation and anchorage-independent growth stimulated by TGF-β.

## Discussion

TGF-β is widely expressed during development to regulate the interactions between epithelial and mesenchymal cells, particularly those in the lung, kidney, and mammary gland. Inappropriate reactivation of EMT during tumorigenesis is now recognized as an important process necessary for the acquisition of invasive and metastatic phenotypes by tumors [1,2]. By cooperating with oncogenes and growth factors, TGF-β potently induces EMT and serves to stabilize this transition by means of autocrine signaling. Moreover, these events seem to underlie the oncogenic activities of TGF-β and its ability to promote cancer progression [20,24-26]. A comprehensive understanding of how TGF-β both suppresses and promotes tumorigenesis remains an unknown and fundamental question that directly affects our ability to effectively target the TGF-β signaling system during the treatment of human malignancies, particularly those of the breast. Indeed, solving this paradox remains the most important aspect of the biological and pathological actions of this multifunctional cytokine.

Despite recent advances in understanding the molecular mechanisms underlying EMT, the question of how to prevent this process effectively in response to TGF-β remains unanswered. We recently discovered that CystC antagonizes TGF-β signaling in normal and cancer cells by interacting physically with TβR-II, thereby preventing TGF-β binding [12]. Importantly, we demonstrated the effectiveness of CystC in inhibiting the invasion of cancer cells and the TGF-β-stimulated invasion of fibroblasts [12]. Because EMT is necessary for the acquisition of invasive and metastatic phenotypes by cancer cells, and because CystC inhibited TGF-β-stimulated invasion, we proposed CystC as a potential antagonist of EMT stimulated by TGF-β.

Accordingly, in this study we show that CystC and Δ14CystC both prevent EMT and its associated increase in MEC motility (Figs 1, 2, 3), as well as antagonizing TGF-β signaling in human breast cancer cells (Figs 4 and 5). We further show for the first time that these CystC molecules inhibit TGF-β signaling in NRK fibroblasts, thus preventing their morphological transformation and invasion through synthetic basement membranes (Fig. 6). Although our understanding of CystC function in regulating TGF-β signaling is in its infancy, our findings suggest that this protease inhibitor might provide an innovative model for the development of novel TβR-II antagonists designed to combat the stimulation of tumor progression and EMT by TGF-β. We further propose that the chemopreventive effectiveness of CystC will be potentiated by its inhibition of cathepsin B-mediated invasion and metastasis [27-30] and its inhibition of the cathepsin B-mediated activation of latent TGF-β [31-33], which co-localizes with cathepsin B to the invading face of malignant tumors [34-37]. Moreover, CystC-mediated cathepsin B inactivation will reduce the activity of the urokinase plasminogen system, which enhances tumor cell extracellular matrix degradation, as well as growth factor and latent TGF-β activation [38]. Cumulatively, the chemopreventive activities of CystC will antagonize cancer cell responses to TGF-β by inhibiting TGF-β binding [12] and by reducing TGF-β bioavailability within tumor microenvironments, thereby alleviating the stimulation of EMT and tumor metastasis in late-stage tumors by TGF-β.

Molecular dissection of TGF-β signaling systems necessary for its induction of EMT has clearly established a role for Smad2/3 in mediating EMT, particularly when coupled with signals emanating from oncogenic Ras [13,39,40]. However, Smad2/3-independent signaling has also been implicated in TGF-β stimulation of EMT. For instance, TGF-β stimulates EMT in cancers of the breast and other tissues by activating phosphoinositide 3-kinase, Akt, RhoA, p160(ROCK), and p38 mitogen-activated protein (MAP) kinase [40-44]. In addition, EMT in TGF-β-treated MECs is abrogated by measures that inhibit β1 integrin activity [42], thus establishing the necessity of β1 integrin expression for EMT stimulated by TGF-β. Finally, by repressing Id2 and Id3 expression [45], inducing Snail and SIP1 expression [46], and stimulating nuclear factor-κB activity [47], TGF-β regulates transcription factor activity operant in mediating the transition from epithelial to mesenchymal cell markers. We show that CystC inhibits the stimulation of Smad2 phosphorylation by TGF-β and the subsequent induction of reporter gene expression in normal and cancer MECs. Thus, reduced Smad2/3 signaling mediated by CystC probably underlies part of its ability to inhibit EMT stimulated by TGF-β. Future studies need to address the role of CystC in regulating Smad2/3-independent signaling stimulated by TGF-β, as well as determining their contribution in preventing the oncogenic activities of TGF-β in human breast cancer cells.

Finally, we were quite surprised to find that CystC, despite its ability to inhibit Smad2/3 signaling, failed to alter the growth-suppressing activities of TGF-β. Although the molecular mechanism(s) underlying this unexpected CystC activity remains to be elucidated, our findings suggest that CystC does not function to abrogate all TGF-β signaling, but may instead specifically alter and/or modulate certain aspects of TGF-β signaling when complexed to TβR-II. In support of this supposition, we find that activation of MAP kinases and Akt by TGF-β requires high cytokine concentrations, whereas that of Smad2/3 requires markedly lower cytokine concentrations (more than 10-fold lower; data not shown). Mechanistically, we propose that maximal stimulation of Smad2/3 by TGF-β requires minimal receptor occupancy (that is, large receptor reserves), whereas maximal stimulation of MAP kinases and AKT requires maximal receptor occupancy (that is, no receptor reserves). Thus, manipulations designed to antagonize the binding of TGF-β to its receptors might elicit disproportionate inhibition of TGF-β signaling systems, resulting in greater inhibition of Smad2/3-independent versus Smad2/3-dependent pathways. Future studies need to address this important question and determine which TGF-β signaling systems are preferentially inhibited by the formation of CystC–TβR-II complexes in normal and cancerous MECs.

## Conclusion

Here we have shown the effectiveness of CystC in inhibiting MEC EMT and fibroblast morphological transformation stimulated by TGF-β, and in antagonizing TGF-β signaling in human breast cancer cells. The ability of CystC to inhibit TGF-β signaling in MECs occurs independently of its inactivation of cathepsin protease activity, presumably by means of CystC–TβR-II complex formation and the prevention of TGF-β binding [12]. We suggest that CystC or its peptide mimetics ultimately hold the potential to improve the therapeutic response of human malignancies regulated by TGF-β, particularly cancers of the breast. Experiments designed to test this hypothesis in animal models of TGF-β oncogenicity and to identify CystC determinants necessary in mediating TGF-β antagonism are currently underway.

## Abbreviations

CystC = cystatin C; EMT = epithelial–mesenchymal transition; GFP = green fluorescent protein; GST = glutathione S-transferase; MAP = mitogen-activated protein; MEC = mammary epithelial cell; NRK = normal rat kidney; TGF-β = transforming growth factor-β; TβR-II = TGF-β type II receptor.

## Competing interests

The authors declare that they have no competing interests.

## Authors' contributions

JPS performed the EMT and morphological transformation studies, as well as acquired and analyzed the data. JRN performed functional analyses of human breast cancer cells, which were generated in part by BJS. WPS conceived of the study, participated in its design, coordination, and data analysis, and drafted the manuscript. All authors read and approved the final manuscript.
